# Estimating loop length from CryoEM images at medium resolutions

**DOI:** 10.1186/1472-6807-13-S1-S5

**Published:** 2013-11-08

**Authors:** Andrew McKnight, Dong Si, Kamal Al Nasr, Andrey Chernikov, Nikos Chrisochoides, Jing He

**Affiliations:** 1 Department of Computer Science, Old Dominion University, Norfolk, VA 23529-0162, USA; 2 Department of Computer Science, Tennessee State University, 3500 John A Merritt Blvd, Nashville, TN 37209, USA

## Abstract

**Background:**

De novo protein modeling approaches utilize 3-dimensional (3D) images derived from electron cryomicroscopy (CryoEM) experiments. The skeleton connecting two secondary structures such as *α*-helices represent the loop in the 3D image. The accuracy of the skeleton and of the detected secondary structures are critical in De novo modeling. It is important to measure the length along the skeleton accurately since the length can be used as a constraint in modeling the protein.

**Results:**

We have developed a novel computational geometric approach to derive a simplified curve in order to estimate the loop length along the skeleton. The method was tested using fifty simulated density images of helix-loop-helix segments of atomic structures and eighteen experimentally derived density data from Electron Microscopy Data Bank (EMDB). The test using simulated density maps shows that it is possible to estimate within 0.5Å of the expected length for 48 of the 50 cases. The experiments, involving eighteen experimentally derived CryoEM images, show that twelve cases have error within 2Å.

**Conclusions:**

The tests using both simulated and experimentally derived images show that it is possible for our proposed method to estimate the loop length along the skeleton if the secondary structure elements, such as *α*-helices, can be detected accurately, and there is a continuous skeleton linking the *α*-helices.

## Background

Over the last ten years, electron cryomicroscopy (CryoEM) experiments yielded increasing numbers of 3D electron density images of protein molecules. The Electron Microscopy Data Bank (EMDB) currently archives the 3D images, referred to as density maps in this paper, with a wide range of resolutions from 3Å to over 80Å [1]. When the density map is resolved to high resolution (3-5Å) [2,3], it is possible to derive the near atomic structure from the density map. However, when the density map is not resolved to the high resolution range, it is still challenging to derive the structure of the imaged molecule [4-6]. Fitting and comparative modeling approaches have been developed to utilize the existing atomic structures in the Protein Data Bank (PDB) [6,7]. These approaches apply when a component of the target density map has been resolved to near atomic resolution structure or when the target protein shares significant homology with existing atomic structures.

**Figure 1:**
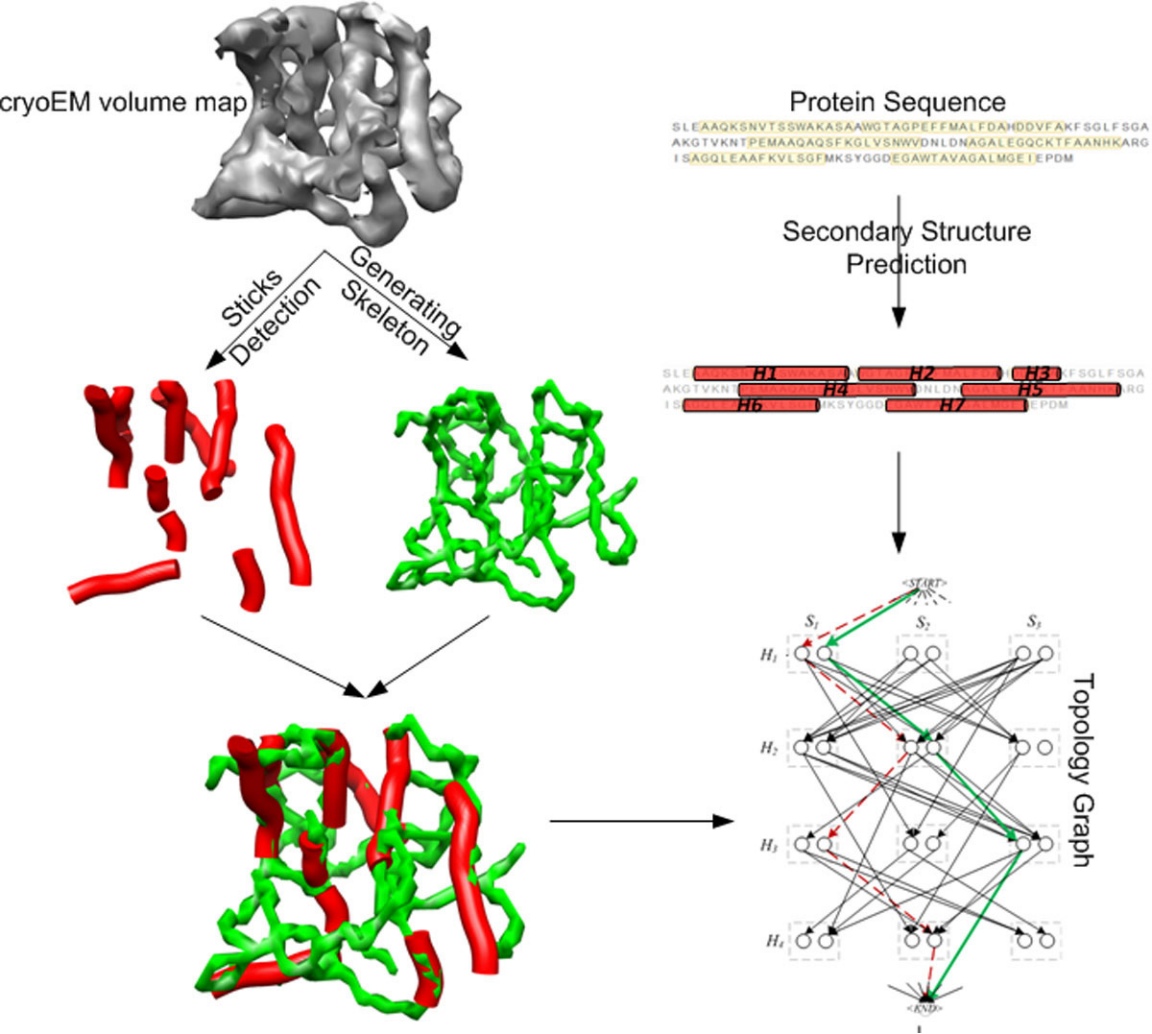
**Deriving the topology of the secondary structure elements from CryoEM images**. The skeleton (green) and detected helices (red) derived from the density map (gray) are combined with the predicted sequence segments of the helices to form a topology graph [8,9,23].

Modeling protein molecules using de novo methods is a general approach to derive the atomic structure from medium resolution (5-10Å) electron density 3D images [6,8-10]. Only the 3D image (top left in Figure 1) and amino acid sequence (top right of Figure 1) are used in de novo processes. It does not need an atomic template protein structure from PDB as required for fitting and comparative modeling methods. First, the secondary structure elements (SSEs) such as *α*-helices (red sticks in Figure 1) and *β*-sheets are often identified using pattern recognition methods [11-16]. Skeletonization methods detect the medial axis (green, left in Figure 1) of a 3D image's iso-surface [10,17]. Next, the amino acid sequence segments (red cylinders, right of Figure 1) of the SSEs can be predicted using existing prediction tools [18-21]. Various approaches have been developed to combine the secondary structure information from the 3D image and 1D sequence in order to derive the topology. The atomic structures can be built once the possible topologies are predicted [6-8].

An amino acid sequence has a direction, starting with a nitrogen atom (N-terminal) and ending with the a carbon atom (C-terminal). The SSE topology is the order in which this sequence traverses the protein's helices and sheets, including the direction of entry into and exit from the secondary structure. The native topology of a protein's SSEs is likely to produce the lowest energy state compared to incorrect topologies [22]. Determining the correct topology is a crucial step in de novo modeling. We have formulated the SSE topology problem into a constrained graph matching problem and provided a dynamic programming algorithm [9]. We later used a dynamic graph approach to handle errors in the data [23].

The distance between two SSEs is an important constraint in graph matching. As an example, two helices closely located in a 3D image should be matched to two helices with similar distance estimated from the 1D sequence. The distance between two ends of two helices (one on each) can be simply estimated as the Euclidean distance [9], or can be measured more accurately along the skeleton [8,23,24]. From the amino acid sequence input, the distance between SSEs can be estimated assuming a 3.8Å distance between adjacent amino acids in the sequence. A scoring function can be developed to represent the overall matching between two sets of SSEs, one from the 3D image and the other from the 1D sequence. The correct topology is assumed to be the one with the best match score.

Despite the relative accuracy of skeletonization algorithms, overestimation may occur if length is measured directly along their piecewise linear curves, which contain many right angles and some error from the thinning process and the 3D image itself.

Here, we extend our previous work in [25], in which we obtained preliminary results testing a computational-geometric method to measure the length of a simplified skeleton. In addition to expanding our test set to include synthetically generated density maps and additional experimentally derived data, we used the directed Hausdorff distance to handle segmentation issues. The measured length appears to agree with the expected length when the SSEs are detected fairly accurately.

## Results and discussion

### Test data and overall process

Two data sets were used in testing performance. The simulated data set consists of fifty randomly selected helix-loop-helix (HLH) motifs from atomic structures in PDB. The proteins extracted exhibit less than 10% sequence identity. Each extracted HLH of the protein structure was used to generate a 3D density map using EMAN1.9 pdb2mrc [26]. The density maps were simulated to 8Å resolution.

The real data set consists of 18 cases whose density maps were downloaded from EMDB with resolution from 4.2Å to 6.8Å. Their EMDB entries are 5030 (6.4Å), 1733 (6.8Å), 5001 (4.2Å), 1740 (6.8Å) and 5168 (6.6Å). Each of these density maps is aligned with their PDB structures at download and provided multiple helix-loop-helix motif samples for the experiment.

The length of a loop was measured along the skeleton voxel points between (and including) the end points of the two surrounding helices. An endpoint of a helix represents an end of the central axis of the helix [11,12]. The helices were detected using *SSETracer*, a simplified version of *SSELearner *[16]. The skeleton was detected using a local maximum clustering method, more details of which are forthcoming in a separate paper. In order to test the accuracy of our algorithm, we visually inspected the detected helices and included only those cases in which the helices were roughly accurate. This was done to distinguish the potential error in our loop length estimation from that of helix detection, skeletonization, or production of the CryoEM image itself.

### Accuracy

**Figure 2:**
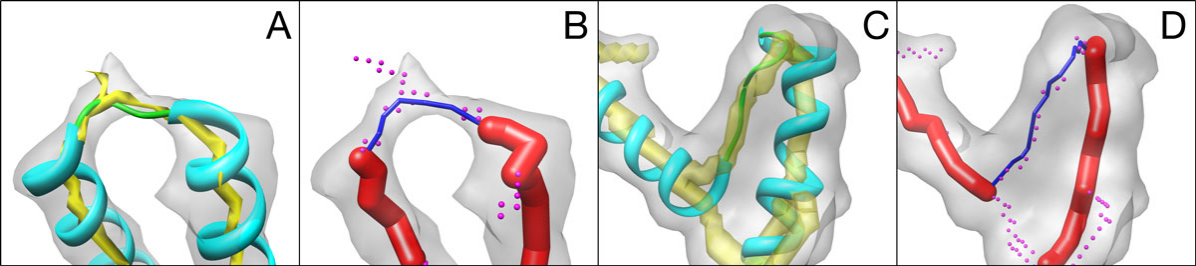
**Loop length estimation from a simplified curve**. The density map (gray), detected helices (red sticks), the true structure (cyan) are shown for the HLH portion of the structure for 1DU0 (PDB Id) in (A, B) and 1MW8 in (C, D). The detected skeleton (yellow) is shown as surface view in (A) and (C), as voxels (red dots) in (B) and (D). The simplified curve derived is shown in blue.

The accuracy of the measurement was evaluated using both the simulated data and the real data from the EMDB. Table 1 summarizes the results for the simulated data. The input to our method includes two pieces of information: the detected helix (red sticks) end points and the skeleton voxels (red dots) (Figure 2B). Each measured length along the skeleton was compared with the expected length of the loop. The expected length was calculated as 3.8Å *×*(*n *+ 1), where *n *is the number of the amino acids on the loop and 3.8Å is the average distance between two amino acids.

**Table 1:** Accuracy of the loop length estimation in the simulated data set.

No	ID	AA	Expected	Measured	Diff	RelErr	DP *∈*
1	1ARO	1	7.6	7.4396	0.1604	2.1	1.00
2	1B0B	1	7.6	7.7384	0.1384	1.8	1.25
3	1BGP	1	7.6	7.6755	0.0755	1.0	1.30
4	1BQB	1	7.6	8.0995	0.4995	6.6	2.30
5	1GUX	1	7.6	7.8102	0.2102	2.8	6.00
6	1B43	2	11.4	11.4264	0.0264	0.2	0.45
7	1B89	2	11.4	11.8811	0.4811	4.2	2.55
8	1BD8	2	11.4	11.3578	0.0422	0.4	0.00
9	1BPY	2	11.4	11.4800	0.0800	0.7	2.25
10	1BR1	2	11.4	11.1461	0.2539	2.2	0.00
11	1FJL	3	15.2	15.4724	0.2724	1.8	1.35
12	1FK5	3	15.2	14.9523	0.2477	1.6	0.00
13	1FUR	3	15.2	15.2643	0.0643	0.4	6.00
14	1H0M	3	15.2	15.3601	0.1601	1.1	2.70
15	1DU0	3	15.2	14.9900	0.2100	1.4	0.60
16	1A87	4	19.0	18.8901	0.1099	0.6	0.95
17	1AIH	4	19.0	19.2057	0.2057	1.1	6.00
18	1AJ8	4	19.0	4.1231	14.8769	78.3	0.00
19	1BMT	4	19.0	19.2313	0.2313	1.2	5.55
20	1BOU	4	19.0	18.9609	0.0391	0.2	0.70
21	1D8L	5	22.8	23.1403	0.3403	1.5	0.60
22	1DI1	5	22.8	22.9243	0.1243	0.5	4.25
23	1DLC	5	22.8	22.5618	0.2382	1.0	0.00
24	1DNP	5	22.8	23.1044	0.3044	1.3	1.70
25	1DP7	5	22.8	22.7786	0.0214	0.1	2.10
26	1CQX	6	26.6	26.2583	0.3417	1.3	0.00
27	1CSH	6	26.6	26.9157	0.3157	1.2	1.85
28	1HM6	6	26.6	7.1461	18.8539	26.3	0.00
29	1MW8	6	26.6	26.2419	0.3581	1.3	0.00
30	1O6L	6	26.6	26.6271	0.0271	0.1	6.00
31	1DJX	7	30.4	30.7842	0.3842	1.3	3.85
32	1E5Q	7	30.4	30.5342	0.1342	0.4	4.65
33	1FFV	7	30.4	30.0703	0.3297	1.1	2.50
34	1H99	7	30.4	30.1897	0.2103	0.7	0.00
35	1IRX	7	30.4	30.7213	0.3213	1.1	6.00
36	1O6L	8	34.2	34.6762	0.4762	1.4	6.00
37	1QVR	8	34.2	34.2838	0.0838	0.2	0.60
38	1S0V	8	34.2	34.2505	0.0505	0.1	0.95
39	1TAU	8	34.2	34.3267	0.1267	0.4	0.70
40	1U09	8	34.2	34.1468	0.0532	0.2	2.05
41	1D6M	9	38.0	38.1574	0.1574	0.4	1.00
42	1FUR	9	38.0	38.3249	0.3249	0.9	2.85
43	1H32	9	38.0	38.1491	0.1491	0.4	0.70
44	1QPC	9	38.0	37.9111	0.0889	0.2	0.00
45	1SU8	9	38.0	37.9337	0.0663	0.2	0.65
46	1QRT	10	41.8	41.7369	0.0631	0.2	0.75
47	1R1H	10	41.8	41.3131	0.4869	1.2	0.00
48	1RJB	10	41.8	41.8528	0.0528	0.1	1.00
49	1XO0	10	41.8	41.8814	0.0814	0.2	1.05
50	2B63	10	41.8	41.4589	0.3411	0.8	4.60

*ID*: PDB ID from which the loop came; *AA*: the number of amino acids in the loop; *Expected *= (*AA *+ 1) * 3.8Å; *Measured*: the estimated length of the loop along the skeleton or its simplification; *Diff*: *Measured - Expected*; *RelErr*: *Difference/Expected*; *DP ∈ *is the value that produced the minimum *Diff *in the estimation.

The fifty tested cases were sorted by the length of the loop, ranging from 1 to 10 amino acids. Almost all the 50 test cases appear to have the error within 0.5Å (column 6 of Table 1). As an example, the loop in 1DU0 (row 15 of Table 1) has three amino acids and the expected length of the loop is 15.2Å. The measured length of the loop along the skeleton is 14.99Å. The relative error is 1.4% of the expected loop length. The simplified curve (blue in Figure 2B) detected by the algorithm appears to be close to the skeleton points (red dots). Another example is from 1MW8 (Figure 2 C, D, row 29 of Table 1) with six amino acids on the loop. The error of the measurement is 0.358Å in this case (column 6 of row 29, Table 1). Note that the skeleton points branch into multiple directions (Figure 2D), yet the algorithm correctly measured the length between the two ending points of the helices by using Hausdorff measurements (see Algorithm). In some cases, as in rows 18 and 28 in Table 1 the greedy step in the Hausdorff computation breaks down and the wrong pair of endpoints was used or the wrong skeleton segment was measured.

**Figure 3:**
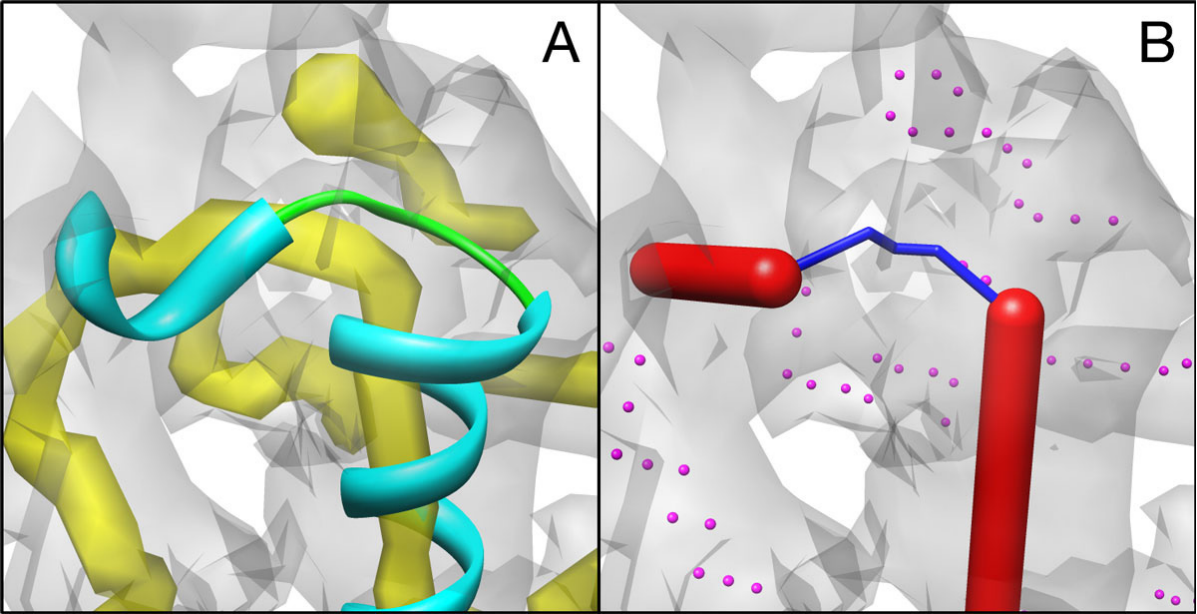
**Detected simplified curve for a loop in CryoEM image (EMDB 5168)**. The color scheme is the same as that in Figure 2.

The test using the experimentally derived density data involves eighteen HLH motifs from density maps with 4-7Å resolution from EMDB. Twelve of the eighteen cases have measured error within 2Å, and six have error between 2Å and 5Å. The real density maps from the experiments are often more challenging with missing density and additional densities that do not align with the true structure. The helices and skeletons detected from the real maps are often less accurate than those from the simulated density maps. Figure 3 shows an example of experimentally derived data in EMDB 5168 (row 15 in Table 2). The difference between the measured and the expected distance is 2.88Å, higher than a comparable case with a synthetic density map used instead. In general, we saw an increase in error using the real density images, due to greater errors in helix detection and skeletonization induced by the noise present.

**Table 2:** Accuracy of the measured loop length for the experimentally derived CryoEM data.

No	ID	AA	Expected	Measured	Diff	RelErr	DP *∈*
1	5030	1	7.6	9.5128	1.9128	25.2	6.00
2	5138	1	7.6	8.2690	0.6690	8.8	6.00
3	5138	2	11.4	11.5490	0.1490	1.3	2.35
4	1733	3	15.2	14.3661	0.8339	5.5	4.05
5	1733	3	15.2	15.0790	0.1210	0.8	3.80
6	5001	3	15.2	11.1189	4.0811	26.8	0.00
7	5001	3	15.2	12.5132	2.6868	17.7	0.00
8	5001	3	15.2	15.6095	0.4095	2.7	2.35
9	5030	3	15.2	15.3747	0.1747	1.1	6.00
10	5030	3	15.2	14.6116	0.5884	3.9	1.75
11	5030	3	15.2	15.1321	0.0679	0.4	3.50
12	5138	3	15.2	14.2916	0.9084	6.0	5.30
13	1733	4	19.0	18.2477	0.7523	4.0	0.00
14	5001	4	19.0	19.1872	0.1872	1.0	6.00
15	5168	4	19.0	21.8790	2.8790	15.2	6.00
16	1740	5	22.8	26.4127	3.6127	15.8	6.00
17	1740	6	26.6	29.3993	2.7993	10.5	6.00
18	5168	6	26.6	22.4231	4.1769	15.7	0.00

See Table 1 for notations except *ID*: the EMDB ID in which the loop was tested.

**Figure 4:**
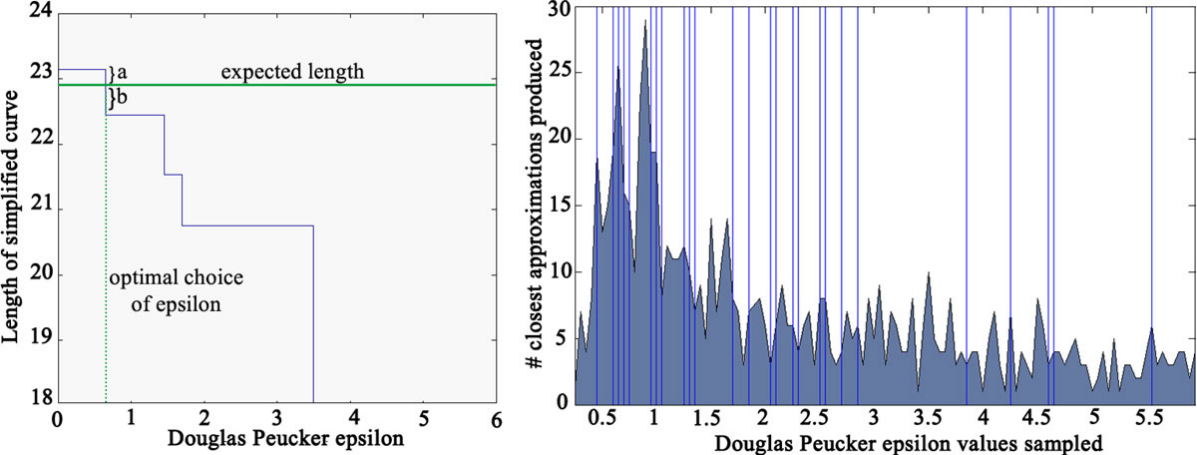
**The Douglas-Peucker ***∈ ***step function**. (Left) The *∈ *step function for case 21 in Table 1 (PDB 1D8L), with the value of *∈ *used for the best estimate. (Right) Distribution of the best *∈ *in the simulated data set of 800 loops. The vertical lines show the values that are listed in Table 1.

The algorithm uses a simplification parameter *∈ *that is user defined. *∈ *is the width of the vertex removal band (refer to the algorithm for more details). In general, the smaller the *∈ *value, the less change in the simplified curve compared to the initial path. In some cases, *∈ *= 0 is the best option, leaving the original path unchanged. In other cases, a much larger value of *∈ *was needed. In order to see the degree of simplification that produced the most accurate results, we sampled *∈*'s range inside the interval 0[6] in increments of 0.05. The measured lengths w.r.t. *∈ *values appear to form a step function, and the value closest to the expected value (Figure 4 left) was marked. As seen from this case, the measured length reduces as *∈ *increases stepwise.

Figure 4 (right) shows the distribution of the values of *∈ *for about 800 simulated cases that had less than 0.5Å difference. The vertical lines represent values of *∈ *for cases in Table 1. It appears that most of the *∈ *values between 0.0 and 1.5 minimize the error in the measurement (Figure 4, right). However, we observed that we need larger *∈ *values for the experimentally derived data than for the simulated density maps. This difference is likely to be associated with the quality of skeletonization and helix detection. For the simulated cases, *∈ *between 0.0 and 1.5 is more likely to produce a good estimate after sufficient preprocessing of the density maps. Multiple *∈ *values might be needed to sample the expected length when working with the experimentally derived cryoEM data.

## Conclusions

We have developed a new approach to estimate loop length along the skeleton from a CryoEM density map. Our tests, using both simulated and experimentally derived images at medium resolution, show that it is possible for our proposed method to estimate fairly accurately the loop length along the skeleton if the SSEs such as *α*-helices and the skeleton are detected fairly accurately.

## Methods

**Figure 5:**
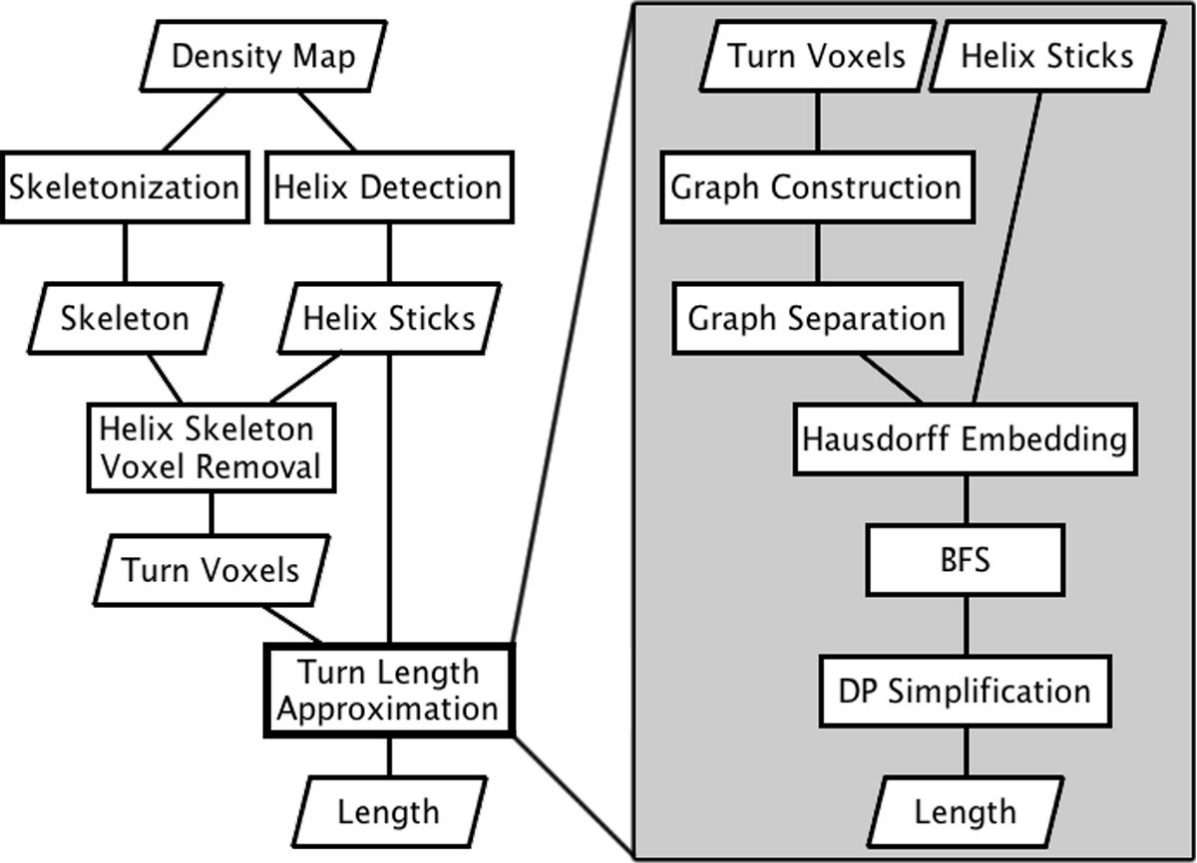
**The process of loop length estimation**. (Left) preprocessing, and (right) the length estimation algorithm.

The overall process to measure the loop length along the skeleton consists of two tasks: preprocessing and length calculation (Figure 5). The purpose of the preprocessing is to derive the skeleton and the endpoints of the two helices from the density map. Once such information is obtained, our algorithm uses graphs and computational geometric concepts to derive the simplified curve.

### Preprocessing

Each case in Table 1 had a density map generated using the HLH segment of the PDB structure and EMAN's *pdb2mrc *[26]. We applied a skeletonization method that utilizes the local maximum points and clustering to derive the skeleton points from the density map. The HLH regions of cases in Table 2 were extracted from entire density images downloaded from EMDB. We used SSETracer, a secondary structure detection method to detect helices from the density map. It is modified from SSELearner [16] with improved speed. Since helix detection is independent of skeletonization, it is necessary to remove the skeleton voxels that belong to the helix region in order to obtain the skeleton belonging to the loop. We removed those skeleton voxels that are within 2.3Å of the central axis of the helix. Note that a helix is 2.3 - 2.5Å in radius [11,27]. After such processing, the skeleton voxels that presumably belong to the loop are segmented from the rest of the skeleton voxels and are subject for length calculation.

### Algorithm

#### Local connectivity graphs

A local connectivity graph (LCG) represents a cluster of skeleton voxels. We impose a constraint on the maximum allowable edge length in a graph, possibly yielding multiple disconnected graphs when all skeleton voxels are considered. For our tests, we normalized the distances between the image's voxels to unity, and chose a maximum edge length *l <*2, producing individual connected subcomponents if they can be clustered into distant groups, referred to as LCGs in this paper.

#### Selecting connected components

Oftentimes, segmented or sparse density data yield multiple LCGs. Also, in general, it is not known which helix endpoints the loop actually lies between. We must then determine the best LCG for each possible pair of helix endpoints. For two helices, one with endpoints *p *and *q *and the other with *r *and *s*, there exists a set *Z *of four possible endpoint pairs: *Z *:= {{*p*, *r*}, {*p*, *s*}, {*q*, *r*}, {*q*, *s*}}. For each endpoint pair *z *∈ *Z*, let the directed Hausdorff distance to an LCG [28] be defined as

(1) $$h\left(z,b\right)=\underset{z_{i}\in z\;\;b_{j}\in b}{\text{max}\;\text{min}}\;d\left(z_{i},b_{j}\right),$$

where *z *is the set of helix endpoints (comprised of voxels denoted *z_i_*) and *b *is an LCG (comprised of voxels denoted *b_j_*) from the set *B *of all LCGs; *d*(*z_i_*, *b_j_*) is then the Euclidean distance between a helix endpoint voxel and LCG voxel. In the presence of multiple LCGs, we choose the best LCG ${\hat{l}}_{z}$ per endpoint pair *z *∈ *Z *by taking the minimum directed Hausdorff distance over all LCGs:

(2) $${\hat{l}}_{z}=\underset{b\;\in \;B}{\text{min}}h\left(z,b\right).$$

We can then use the voxels of ${\hat{l}}_{z}$ to build our model of the loop between the endpoints of *z*.

**Figure 6:**
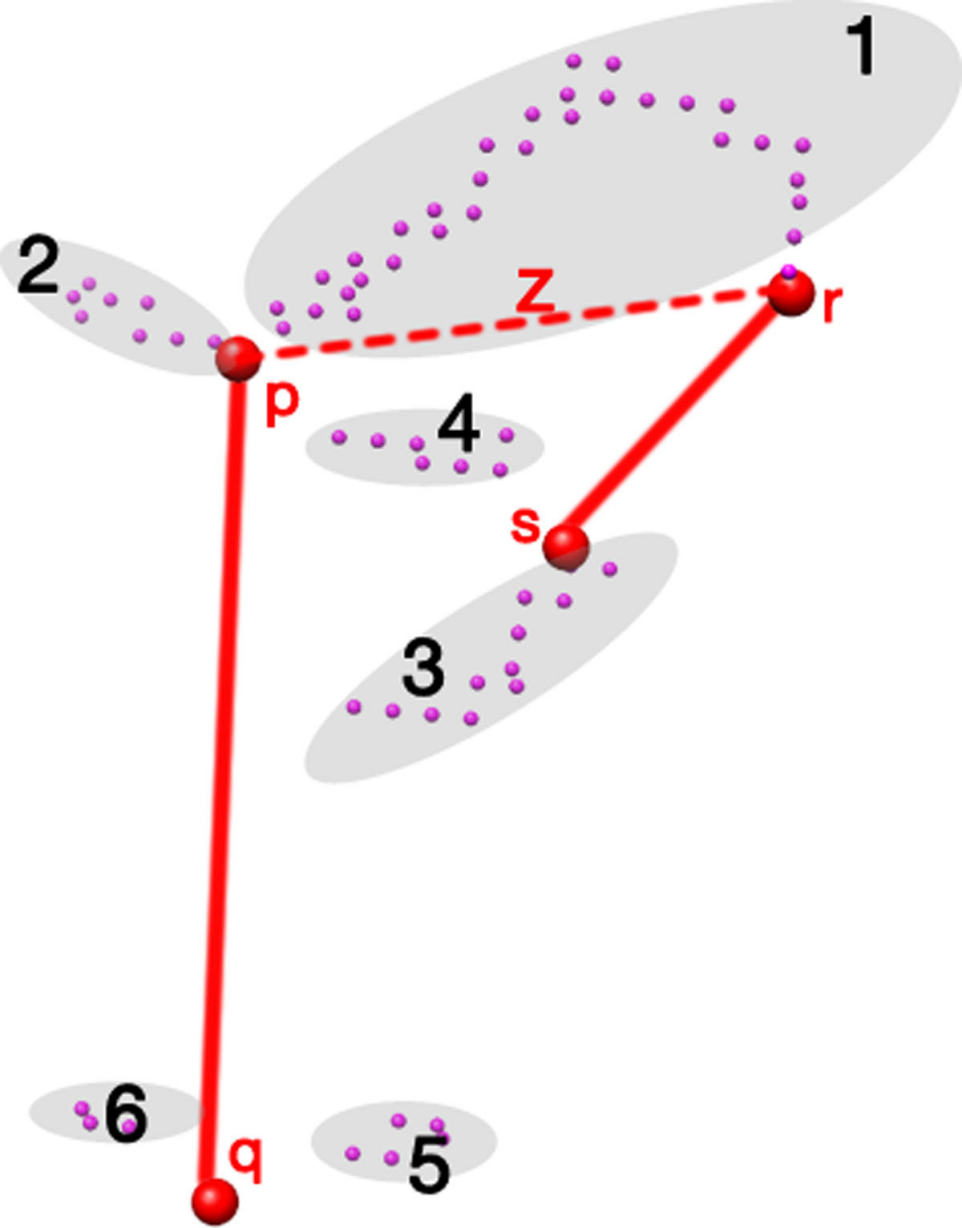
**Hausdorff distance comparison of the connected skeleton point groups**. Two detected helices (solid red lines), with a pair *z *of helix endpoints (connected by the red dashed line) and several LCGs (gray ellipses) from PDB 1O6L. In this case, LCG *1 *is closest to *z *in terms of directed Hausdorff distance.

It should be noted here that the directed Hausdorff is not commutative-in general, *h*(*M*, *N*) ≠ *h*(*N*, *M*)- and we always chose *M *as a set (pair) of helix endpoints, and *N *as an LCG. Figure 6 shows the configuration for case 30 (PDB 1O6L) from Table 1, where we want to find ${\hat{l}}_{z}$ among the set of LCGs *B *:= {1, 2, 3, 4, 5, 6} to search for the loop that *may *lie between the helix endpoint pair *a*. After finding ${\hat{l}}_{z}$ using equation (2), we repeat the procedure for each other helix endpoint pair. We try connecting the helix endpoints to their respective closest voxels in ${\hat{l}}_{z}$ with respect to the Euclidean distance. If either of the new edges connecting *p *or *r *is longer than 5Å, we discard the combination as an infeasible path.

#### Pathfinding

After finding the best LCG for a given possible helix endpoint pair, the next step is constructing a path that traverses it in a way that will approximate the loop. We simply performed a breadth-first search starting from one of the helix endpoints we added, and reconstruct the path that ends at the other one in the graph [29], with a helix endpoint as the source. For a given HLH, we find four such paths, one for each possible helix endpoint pair.

#### Path simplification

Ideally, the distance between two specific ends of two helices should be measured along the skeleton connecting the two ends by using our initial path. If we simply add the length of the line segments along the initial path, there is a danger of over estimation due to the potential zigzagging induced from drawing a path along the edges of the cubic lattice of the 3D image.

**Figure 7:**
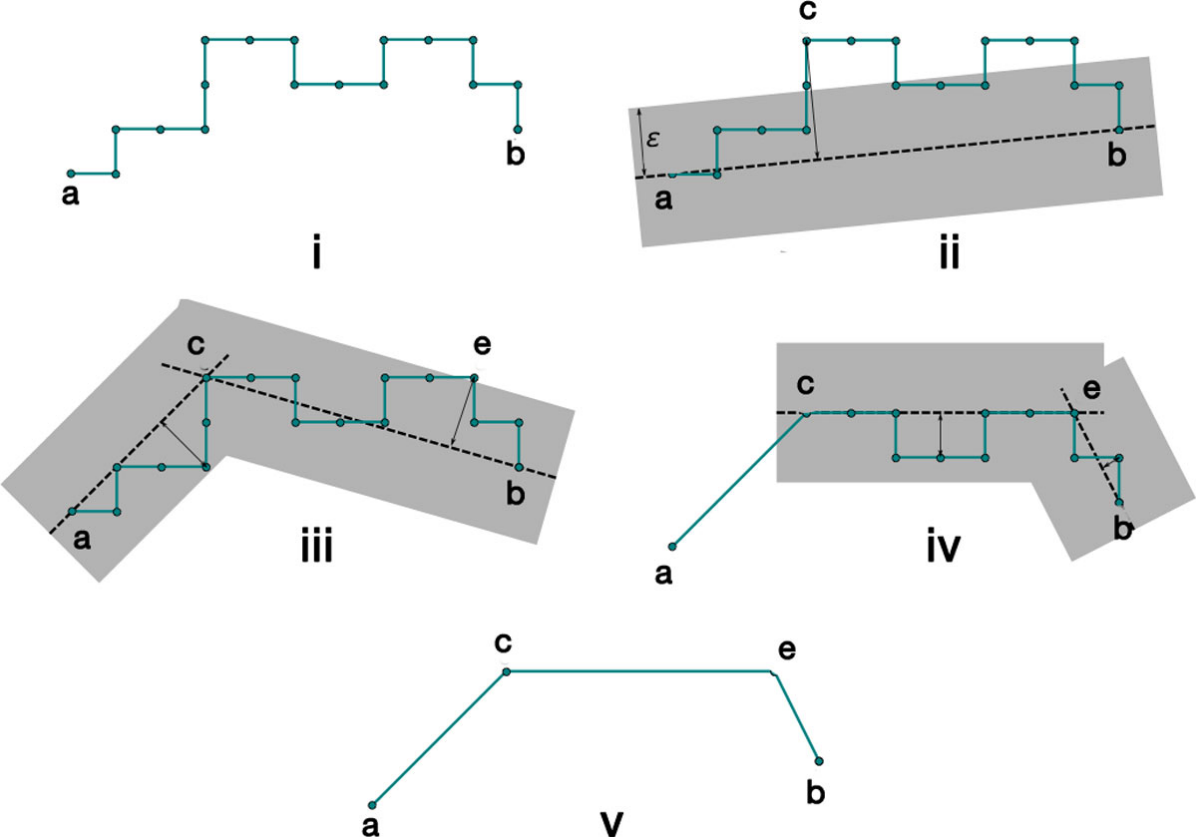
**Recursive iterations of the Douglas Peucker line simplification algorithm**. Each gray region, as in (ii), illustrates the distance from the test line ($\bar{ab}$in (ii)) defined by *∈*.

Douglas-Peucker line simplification [30,31] is the systematic removal of points that lie beyond some distance *∈ *from a line describing the general orientation of a piecewise linear curve (polyline) or one of its subsegments. Consider the two-dimensional example in Figure 7. Part (i) shows an initial polyline $\bar{a...b}$. The algorithm is recursive, and takes as parameters the tolerance *∈ *(Figure 7 (ii)) and a multi-point segment of a polyline. At each recursive iteration it finds an interior point of the current segment which is the most distant from the straight line connecting the end points of the segment, as in Figure 7 (ii) and 7(iii). If all of the current segment's vertices lie within the *∈ *band, the segment is replaced with a straight line segment containing only its endpoints. Otherwise, the segment is split at the most distant point and each subsegment is handled recursively. In Figure 7 (iii), $\bar{ac}$ and $\bar{cb}$ are treated in different recursive calls; *e *is the farthest point from $\bar{cb}$, and no points lie outside the epsilon band for $\bar{ac}$. Overall, the initial polyline $\bar{a...b}$ is simplified into polyline $\bar{aceb}$, which approximates the length of the loop between helix endpoints.

## List of abbreviations

CryoEM: electron cryomicroscopy; SSE: secondary structure element - either *α*-helices or *β*-sheets; EMDB: Electron Microscopy Data Bank; PDB: Protein Data Bank; HLH: helix-loop-helix motif found in protein structures; LCG: local connectivity graph - a connected graph of skeleton voxels with a maximum allowed edge length.

## Competing interests

The authors declare that they have no competing interests.

## Authors' contributions

Andrew McKnight compiled the test set and developed the software for the algorithm. Jing He, as advisor, guided through the problem she has previously researched regarding SSE topology matching. Nikos Chrisochoides and Andrey Chernikov provided technical information and guidance for our algorithm. Dong Si and Kamal Al Nasr provided software and technical support for SSE extraction from and skeletonization of cryoEM density maps.

## Declarations

Research was funded in part by NSF grants: CCF-1139864, CCF-1136538, and CSI-1136536 and by the Richard T. Cheng Endowment, the ODU MSF fund and the ODU startup fund. Nikos Chrisochoides helped defray publication costs via NSF funding.

This article has been published as part of *BMC Structural Biology *Volume 13 Supplement 1, 2013: Selected articles from the Computational Structural Bioinformatics Workshop 2012. The full contents of the supplement are available online at http://www.biomedcentral.com/bmcstructbiol/supplements/13/S1.
